# Design and rationale of a randomized, placebo-controlled trial on the efficacy and safety of sulodexide for extended treatment in elderly patients after a first venous thromboembolism

**DOI:** 10.1007/s11739-020-02381-5

**Published:** 2020-05-25

**Authors:** Gualtiero Palareti, Cristina Legnani, Emilia Antonucci, Serena Zorzi, Angelo A. Bignamini, Corrado Lodigiani, Alberto Tosetto, Lorenza Bertù, Vittorio Pengo, Sophie Testa, Walter Ageno, Domenico Prisco, Paolo Prandoni, Daniela Poli

**Affiliations:** 1 Arianna Anticoagulazione” Foundation, Via Paolo Fabbri 1/3, 40138 Bologna, Italy; 2 Department of Pharmaceutical Sciences, School of Specialization in Hospital Pharmacy, University of Milan, Milan, Italy; 3 Thrombosis and Hemorrhagic Diseases Unit, Humanitas Research Hospital and Humanitas University, Rozzano (Milan), Italy; 4 Divisione Di Ematologia, Centro Malattie Emorragiche E Trombotiche, AULSS8 Berica, Vicenza, Italy; 5 Centro Di Ricerca Sulle Malattie Tromboemboliche E Le Terapie Antitrombotiche, Università Degli Studi dell’Insubria, Varese, Italy; 6 Thrombosis Research Laboratory, Department of Cardiac-Thoracic-Vascular Sciences and Public Health, University of Padua, Padua, Italy; 7 Department of Laboratory Medicine, Haemostasis and Thrombosis Center, AO Istituti Ospitalieri, Cremona, Italy; 8 Dipartimento Di Medicina E Chirurgia, Università Degli Studi Dell’Insubria, Varese, Italy; 9 DMSC Università Di Firenze, SOD Medicina Interna Interdisciplinare, AOU Careggi, Florence, Italy; 10 Centro Trombosi, Azienda Ospedaliera Careggi, Florence, Italy

**Keywords:** Elderly, Extension, Recurrence, Sulodexide, Venous thromboembolism

## Abstract

How to prevent recurrences after a first venous thromboembolic (VTE) event in elderly patients is still an open issue, especially because of the high bleeding risk of anticoagulation in these patients. The placebo-controlled “Jason” study aims at assessing the efficacy and safety for secondary VTE prevention in elderly patients of oral Sulodexide (Vessel^®^) administration, a mixture of glycosaminoglycans (Alfasigma, Bologna, Italy) which proved effective against recurrences in a general population (SURVET study) without major bleeding (MB) complications. 1450 patients, aged ≥ 75 years, after at least 3 months of anticoagulation treatment for a first VTE episode, are double-blind randomized to receive for 12 months either sulodexide 500 lipasemic units (LSUs) twice daily, or sulodexide 250 LSU twice daily + indistinguishable placebo, or indistinguishable placebo. Primary outcomes for efficacy are the composite of death for VTE and recurrent VTE, and occurrence of MB for safety. Secondary outcomes include stroke, cardiovascular death and other thromboembolic events, and MB + clinically relevant non-MB. The first patient is scheduled to be randomized in May 2020. The study protocol has been approved by AIFA (Agenzia Italiana del Farmaco) and the Ethics Committee of the coordinating center. Written informed consent will be obtained from all patients prior to study participation. Jason study is an investigator-initiated trial, promoted by “Arianna Anticoagulazione” Foundation, Bologna, Italy, and supported by Alfasigma, Bologna, Italy. Study findings will be disseminated to participant centers, at research conferences and in peer-reviewed journals. Trial registration numbers NCT 04257487; EudraCT (2019–000570-33).

**Electronic supplementary material:**

The online version of this article (10.1007/s11739-020-02381-5) contains supplementary material, which is available to authorized users.

## Introduction

Venous Thromboembolism (VTE), encompassing deep vein thrombosis (DVT) and/or pulmonary embolism (PE), is a frequent and severe disease with an incidence of 1 to 2 per 1000 persons/year [1, 2]. The risk of VTE is definitely higher in the elderly population (i.e., older than 75), with an incidence that may reach 0.5 per 100 persons/year [3]. Following a first VTE event, anticoagulation therapy for 3–6 months is the mainstay treatment for all patients as an initial and long-term therapy. The extension of anticoagulation beyond this period (extended therapy) is suggested in patients with a high risk of VTE recurrence, provided that the risk of bleeding during anticoagulant therapy is not high, as assessed on individual basis [4]. Among conditions increasing the risk of bleeding there is the elderly population, either because advanced age is in itself a high-risk factor of bleeding and also for the more frequent presence of comorbid conditions and associated treatments that may increase the bleeding risk in this population.

It would be important to identify those elderly subjects who may benefit from extending anticoagulation beyond the first three months, and limit it to these patients; however, this is not an easy task to achieve. Indeed, the incidence of recurrent VTE was not found to differ significantly between patients with unprovoked and provoked events in a cohort of elderly patients [5]. In addition, the measurement of D-dimer levels, which may help identify patients requiring an extended anticoagulation [4, 6], is unlikely to predict the risk of recurrence in elderly patients, probably as it is commonly altered in this population [7]. Finally, the presence of inherited thrombophilic alterations was shown to be relatively frequent in elderly people with VTE, but not associated with the risk of VTE recurrence [8].

According to the latest American College of Chest Physician—ACCP guidelines [4] anticoagulation therapy should be discontinued after a 3–6-month treatment in all patients aged 75 years or older with a first VTE event, even if additional risk factors of bleding are not present. Whether the administration of low-dose rivaroxaban or apixaban, which proved to be effective and safe for the long-term treatment of most patients with a first VTE episode [9, 10], can be recommended also for elderly patients is uncertain, because of the low prevalence of these patients in both clinical trials (between 11 and 13% of the patients included). Furthermore, the results of these trials obtained in the elderly population, both in terms of efficacy and safety, were certainly less good and substantially less satisfactory than in young patients.

Sulodexide (Vessel^®^), a highly purified mixture of glycosaminoglycans (Alfasigma, Bologna, Italy), is an antithrombotic compound that has recently been used in the international, randomized, placebo-controlled SURVET study [11]. This study showed that treatment for 2 years with oral Sulodexide Vessel^®^ (500 LSU, BID) in patients who had suffered from a first idiopathic VTE and had already undergone an adequate period of anticoagulant therapy reduces the risk of thrombotic recurrence by 50% compared to patients receiving placebo, without involving any case of major bleeding.

**Fig. 1:**
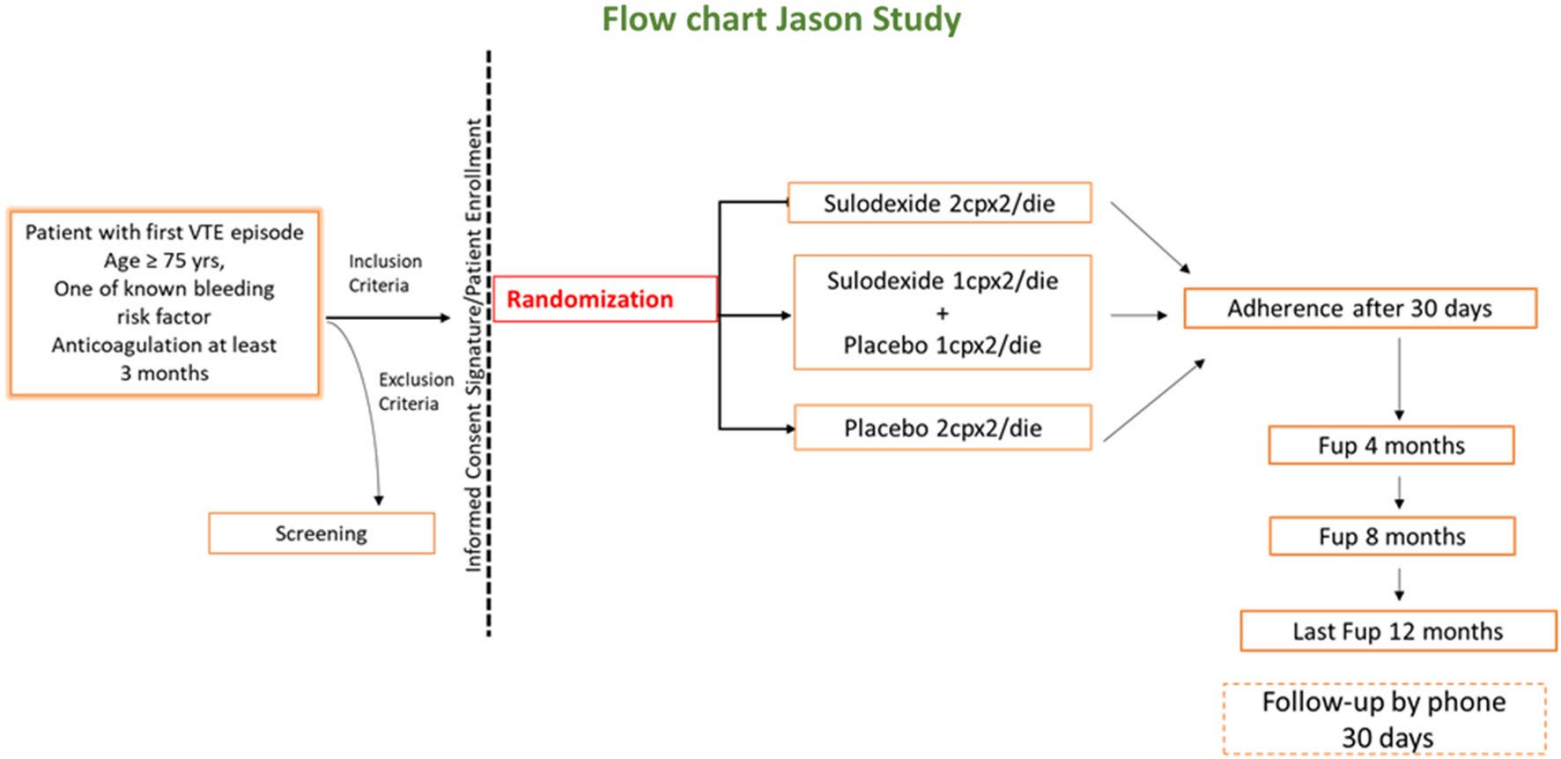
Flow chat Jason study

In the Jason study we want to assess the efficacy to prevent recurrent VTE events and the safety of two different doses of oral Sulodexide (500 LSU BID and 250 LSU BID), compared with placebo in elderly patients (≥ 75 years) who also have at least one clinical condition generally considered as a risk factor for bleeding. Patients should have had a first VTE, and have completed at least 3 months of anticoagulation therapy of any type. Additional aims of the study are to determine whether the lower dose of sulodexide is as effective as the higher one, and to examine the effect of treatment on cardiovascular deaths and on arterial thrombotic outcomes (Fig. 1).

## Method and analysis

### Primary and secondary objective

The primary efficacy objective is the composite of recurrent VTE episodes (proximal DVT and/or PE) and VTE-related mortality. The primary safety endpoint is the incidence of major bleeding (MB), according to the definition by the International Society on Thrombosis and Haemostasis [ISTH] criterion (Table 1) [12]. Secondary endpoint for efficacy is the composite of hospitalizations and deaths due to cardiovascular events (acute myocardial infarction, ischemic stroke). Secondary safety endpoint is the cumulative incidence of MB and non-major but clinically relevant hemorrhages [NMCRB] (Table 1) [13].

**Table 1:** Criteria for definition of bleeding events

(1) Major bleeding (MB) [12]
Acute clinically overt bleeding accompanied by one or more of the following:
Fatal bleeding
Bleeding that occurs in at least one of the following critical sites: intracranial, intraspinal, intraocular, pericardial, intra-articular, intramuscular with compartment syndrome, retroperitoneal
A decrease in hemoglobin of 2 g/dl or more
A transfusion of 2 or more units of packed red blood cells
(2) Clinically relevant non-major bleeding (CRNMB) [13]
Any sign or symptom of hemorrhage (e.g., more bleeding than would be expected for a clinical circumstance, including bleeding found by imaging alone) that does not fit the criteria for the ISTH definition [12] of major bleeding but does meet at least one of the following criteria:
Requiring medical intervention by a healthcare professional
Leading to hospitalization or increased level of care
Prompting a face to face (i.e., not just a telephone or electronic communication) evaluation
(3) Minor bleeding (MinB)
All acute clinically overt bleeding events not meeting the criteria for either major bleeding or clinically relevant non-major bleeding were classified as minor bleeding

### Patients, study design

The Jason study is a phase III, investigator-initiated, multicenter, randomized to parallel groups, placebo-controlled, double-blind and non-commercial (no-profit) trial conducted in 48–53 Italian study sites (see appendix A). Patients eligible for the study are: those aged ≥ 75 years at screening, with at least one factor for bleeding during anticoagulation (Table 2 lists the inclusion and exclusion criteria), who have had a first episode of lower extremity proximal DVT and /or PE, that was idiopathic or associated with weak or removed risk factors, and have completed a period of anticoagulant treatment (regardless of the drug used) of at least 3 months. Prior to enrollment, the investigators obtain informed consent from the patients, recommend discontinuing the ongoing anticoagulant therapy, ask each patient to fill a self-assessment questionnaire for the Villalta score [14] both at the beginning and at the end of treatment, and fill-in the enrolment forms in the electronic database.

**Table 2:** Inclusion and exclusion criteria

Inclusion criteria
Patients of both sexes aged ≥ 75 years at the time of enrolment
With a first event of proximal lower extremity DVT and / or PE, idiopathic or associated with weak or removed risk factors
With at least one of the known risk factors for bleeding listed below:
Hypertension
Renal failure
Thrombocytopenia
Diabetes
Antiplatelet therapy (ASA maximum 140 mg/die)
Frequent falls (> 2 /years)
Nonsteroidal anti-inflammatory drug
Liver failure
Previous Stroke
Anemia
Poor anticoagulant control
Alcohol abuse
Who at the time of enrolment have already undergone a period of anticoagulant therapy (whatever the medication used) of at least 3 months and the therapy has not been suspended for more than 30 days
Without other clinical indication for anticoagulation
Capable and able to provide informed consent
Exclusion criteria:
Patients aged < 75 years at the time of the recruitment visit
With a "provoked" index event, which occurred:
Within 3 months of major surgery or trauma
After bed rest > 4 days
Cast/immobility within 3 months
With severe PE as index event, life-threatening or treated with thrombolytic therapy
Index event represented by isolated distal DVT or superficial venous thrombosis
Thrombotic event in sites other than the deep proximal veins of the lower limbs
Anticoagulant therapy for less than 3 months at the time of enrolment
Discontinuation of anticoagulant therapy for > 30 days at the time of enrolment
Recurrent episodes of DVT or PE
Presence of severe post-thrombotic syndrome (Villalta score > 15 or venous ulcer)
Presence of other clinical conditions requiring anticoagulant therapy
Active cancer
Presence of Inferior vena cava filter
Known bleeding diatheses
Treatment with antiplatelet drugs other than ASA; ASA is allowed up to 140 mg/day
All clinical conditions requiring long-term treatment with low molecular weight heparin (LMWH)
Antiphospholipid Antibody Syndrome
Presence of serious thrombophilic alterations
Presence of chronic diseases in acute or active phase (e.g.: inflammatory bowel disease)
Cardiorespiratory failure (NYHA class 3 or 4)
Patients incapacitated or refusing to sign the informed consent
Patients with life expectancy < 1 year
Patients residing in a disadvantaged geographical area
Patient already enrolled in other clinical trials
Patients with > systolic pulmonary artery pressure (> 40 mmhg, upper limit for elderly)
Contraindication to Sulodexide (VESSEL^®^)

A total of 1450 patients is planned to be enrolled in the study and randomized into three treatment arms: A: Sulodexide Vessel^®^, 2 capsules of 250 LSU BID for 12 months; B: Sulodexide Vessel^®^, 1 capsule of 250 LSU plus 1 capsule of matching placebo BID for 12 months; and C: 2 matching placebo capsules BID for 12 months. The assigned treatment is then given to the patient; after one month the patient is seen again in the outpatient clinic to reinforce his comprehension of the study modality, adhesion to treatment and answer his doubts. The control visits are performed at the 4th and 8th month after the initiation of treatment; at these visits, the study material for the subsequent period is supplied, the general clinical conditions, possible complications, new events or diseases as well as adverse events (AEs) are recorded in the electronic case-report-form (eCRF), and the remaining study material is collected to assess compliance. All AEs, defined as any untoward medical occurrence or worsening of a pre-existing medical condition in patients receiving the study drug (sulodexide or placebo) and that may or not have a causal relationship with the study drug, which occur from screening to end of the study will be collected and seriousness, severity and causality will be assessed by the investigators. The Pharmacovigilance for the study will be accomplished by Dr. Elisabetta Bigagli, from the “Dipartimento di Neuroscienze, Psicologia, Area del Farmaco e Salute del Bambino, NEUROFARBA”, University of Florence, Italy (farmacovigilanza-noprofit@neurofarba.unifi.it).

One month after the last visit (at 12th month of treatment), each patient will be contacted by telephone to assess the clinical conditions after treatment withdrawal. Patients are instructed to contact the clinical center immediately if symptoms developed suggestive of VTE or in case of bleeding.

In cases of a suspected recurrent DVT, the results of compression ultrasonography (CUS) are compared with those of the last available previous examination. A recurrent DVT is diagnosed if a previously fully compressible segment (contralateral or ipsilateral) is no longer compressible or if an increase of at least 4 mm in the diameter of the residual thrombus during compression was detected [15]. In patients with suspected PE, recurrence will be diagnosed on the basis of objective algorithms [16, 17] incorporating clinical probability, helical CT (or ventilation-perfusion lung scanning), CUS, and/or D-dimer testing as appropriate. All suspected outcome events and deaths will be evaluated by a central adjudication committee whose members are unaware of the patient’s name, the center where the patient had been enrolled, and the type of treatment assigned.

### Data management

The local investigators will record anonymised patients’ data on the eCRF. All study-related information about participants will be stored securely and kept strictly confidential. All study sites will be supervised by a dedicated remote monitor throughout the entire study duration. Before enrolment of the first patient, the investigators active in each site will have a telephone training session with the study monitor to clarify the design of the study, the knowledge of the protocol, the correct eCRF use, and assure the medical and formal instruction of investigators. The study monitor will check the adhesion to the study procedures and the complete and correct entry into the eCRF.

Treatment with the study medications will be permanently discontinued in case of one of the following events: serious adverse events, occurrence of a pathology requiring anticoagulant drug treatment for an indefinite period of time, onset of cancer or other serious pathology, withdrawal of the informed consent, decision of the attending physician based on the patient's clinical needs. A temporary discontinuation of treatments does not imply premature termination of the study. Each treatment may be temporarily discontinued in case of surgery, invasive procedure, or during treatment with LMWH. The period of interruption will not imply variations of duration in the observation. A period of temporary discontinuation not exceeding 20 days (even non-consecutive) for every 4 months of participation in the study is acceptable for the continuation of the study,

### Early termination or discontinuation of the study

The promoter may interrupt the study at any time if recommended by the "Data Safety and Monitoring Board" (DSMB) following the assessment of the results of the scheduled interim analyses. The DSMB will examine the available data when 30% and 60% of the total events planned for the study, or 30% and 60% of the patients concluded the study, whichever comes first. The DSMB will then inform the promoter if conditions of evident superiority, evident inferiority or futility exist for the entire study or for one of the arms in the study. The recommendation may assume the form of early interruption of the entire study; early interruption of one of the arms; continuation as expected. Furthermore, the DMSB may also recommend—with adequate justification—the redistribution of subsequent cases between the study arms or even the change in sample size. In the event of early termination of the study or interruption of an active arm, the promoter will promptly notify the competent authorities, investigators and ethics committees.

### Sample size calculation

Based on available data, the incidence of the primary endpoint for efficacy is estimated to be 13 per 100 patient-years. Available data (study SURVET [11]) indicate that the relative risk reduction (RRR) of events in the high-dose group (500 U BID) should be approximately 0.50. It can be expected that the RRR in the low-dose group (250 U BID) is approximately 0.25. The total relative risk reduction among treated subjects can, therefore, be estimated at approximately 0.38. The expected incidence of events among all examined patients should, therefore, be approximately 8 per 100 patient-years.

The incidence of major bleeding (co-primary endpoint) can be estimated as approximately 1 per 100 patient-years and is not modified by the study treatments.

The following efficacy of hypotheses can be formulated:

- hierarchically superior hypothesis: ${H}_{0}: {\pi }_{\mathrm{t}\mathrm{r}\mathrm{e}\mathrm{a}\mathrm{t}\mathrm{e}\mathrm{d}}={\pi }_{\mathrm{c}\mathrm{o}\mathrm{n}\mathrm{t}\mathrm{r}\mathrm{o}\mathrm{l}\mathrm{s}}; {H}_{A}: {\pi }_{\mathrm{t}\mathrm{r}\mathrm{e}\mathrm{a}\mathrm{t}\mathrm{e}\mathrm{d}}\ne {\pi }_{\mathrm{c}\mathrm{o}\mathrm{n}\mathrm{t}\mathrm{r}\mathrm{o}\mathrm{l}\mathrm{s}}$ regardless of dose, where the size of the minimum detectable difference is that indicated above;
- hierarchically inferior hypothesis (to be examined if the previous null hypothesis is rejected):
  ${H}_{0}: {\pi }_{250x2}-{\pi }_{500x2}\ge \delta ; {H}_{A}: {\pi }_{250x2}-{\pi }_{500x2}<\delta$, where *δ* (value expressing the acceptable non-inferiority limit) is set at 0.045. This indicates that the outcome with the low dose is considered not inferior to that of the higher dose if the proportion of events is not greater than that of the high dose increased by 4.5 percentage points.

The only safety hypothesis considered is that the incidence of MB events among treated patients (regardless of dose) is not superior to that among controls and, furthermore, the higher limit of the 95% confidence interval of incidence does not reach 3%. The hypotheses can, therefore, be expressed as: ${H}_{0}: {\pi }_{\mathrm{t}\mathrm{r}\mathrm{e}\mathrm{a}\mathrm{t}\mathrm{e}\mathrm{d}}-{\pi }_{\mathrm{c}\mathrm{o}\mathrm{n}\mathrm{t}\mathrm{r}\mathrm{o}\mathrm{l}\mathrm{s}}\ge \delta ; {H}_{A}: {\pi }_{\mathrm{t}\mathrm{r}\mathrm{e}\mathrm{a}\mathrm{t}\mathrm{e}\mathrm{d}}-{\pi }_{\mathrm{c}\mathrm{o}\mathrm{n}\mathrm{t}\mathrm{r}\mathrm{o}\mathrm{l}\mathrm{s}}<\delta$, where *δ* is equal to 0.02. This means that the outcome among treated is considered not inferior compared to that among controls, in case it results in a proportion of hemorrhagic events non greater than that among controls increased by 2 percentage point and, in any case, with an upper limit of the 95% confidence interval not greater than 3%.

The two co-primary endpoints are not correlated consequently do not influence multiplicity. Two other items influence multiplicity: the analyses on the primary efficacy endpoint and the analyses *ad interim* to be supplied to the DMSB of the study, that overall imply a decrease of the critical alpha value of the final analysis of the primary efficacy endpoint from 0.050 to 0.048 two-sided, and of the co-primary safety endpoint from 0.0500 to 0.0465 one-sided. Consequently:

- (a) at least 460 patients per group (460 controls and 920 treated) have 80% power to observe with 95.2% confidence (alpha = 0.048 two-tailed) a difference of incidence between treated and controls equal to approximately 5 percentage points, on the assumption of an incidence among controls of 13% [18].
- (b) the same number is sufficient to have 80% power to observe with 95.2% confidence (alpha = 0.048 one-tailed) the non-inferiority of the low-dose vs. the high-dose group, on the assumption of an incidence of events of approximately 10% in one group and 6% in the other, accepting a non-inferiority margin of 4.5 percentage points [19].
- (c) the same number is largely sufficient to have 90% power to observe with 95.35% confidence (alpha = 0.0465 one-sided) the non-inferiority of treated groups combined vs. controls, on the hypothesis of an incidence of hemorrhagic events of 1% among controls and accepting a non-inferiority margin of 2 percentage points [19]and, anyway, accepting an upper limit of the one-sided 95% confidence interval of incidence—determined by exact binomial test—not greater than 3%.

The total sample size should, therefore, be of approximately 1380 valid cases. Accounting for approximately 5% not assessable cases, the total number of subjects to be recruited should be of approximately 1450 (theoretically, 1464).

### Statistical plan

All randomized subjects will be examined for efficacy and safety. Subjects who have been randomized to treatment but refuse to receive the assigned medication are classified as screening failures and excluded from all analysis. The safety analyses shall be performed on all randomized subjects; the efficacy analyses will be performed on randomized subjects (Intent-To-Treat population; ITT). Subjects presenting violations of the admission criteria, such to make the individual case inappropriate for the study, will be excluded as well as the “not assessable” cases, that is, those subjects who had been properly followed during the whole observation period, had the final evaluation but this evaluation was inconclusive as to the efficacy endpoints and cannot be repeated. Exclusion of a subject from the ITT population will be decided jointly in a blind way by the Steering Committee and the Adjudication Committee. The resulting population (modified ITT; mITT) will be the population submitted to the efficacy analyses.

Furthermore, the Steering Committee will identify the subjects, among the mITT population, for whom the Adjudication Committee will have validated one of the primary endpoints or who have reached the end of the observation without primary endpoints, with a compliance of at least 75%. This population (per protocol, PP) will be used for sensitivity analyses of the primary efficacy and safety endpoints.

Data collected at recruitment will be analyzed on the mITT population and, only if necessary, also on the safety and PP populations. Collected data for continuous variables will be described as mean with standard deviation and 95% confidence interval if normally distributed, or as median with range interquartile and 95% CI in case of deviation from a normal distribution. Nominal variables will be described as absolute and relative frequency tables. Statistical analysis of the baseline variables [analysis of variance (ANOVA) complemented with the Tukey and Dunnet tests, or Kruskal–Wallis test, as appropriate, and chi-square test for nominal variables] is planned for those variables that at the descriptive analysis, will suggest potential differences of clinical relevance.

The primary endpoint events will be expressed as frequencies and as a time to the event occurrence. The primary analysis of the events will be the survival analysis according to Cox, using the treatment as a covariate to estimate the hazard ratio, the relevant 95% confidence interval and the associated P value; other covariates may subsequently be analyzed [age (in decades), sex, the type of index event (PE—alone or associated with DVT—or DVT alone), the duration of exposure to anticoagulation (< 6 months / ≥ 6 months) and, if appropriate, the level of compliance (< 75%/ ≥ 75%)]. This analysis will be applied to the mITT population and, for what applicable, to the PP population.

The results of the primary efficacy endpoint will also be examined as frequencies. Since it is possible that in the mITT population there will be censored cases (those who left prematurely the study without having reached an endpoint), procedures will have to be applied to replace the missing outcome. Two procedures are anticipated: (a) a “worst case” classification, in which all censored cases will be classified as a failure (endpoint reached); (b) if appropriate, attribution to the missing outcomes of the outcome seen in the nearest-neighbor case, estimated by the propensity score computed using the same predictors used for the Cox model, with and without the predictor “treatment”. These results will be analyzed with the exact Fisher’s test, estimating the relative risk and the relevant 95% confidence interval. The primary safety endpoint (severe hemorrhages) will be analyzed in the same way as the primary efficacy endpoint, using however one-sided tests in accordance with the non-inferiority hypothesis expressed in the sample size calculation.

The secondary endpoints will be examined on the mITT population without replacement of missing data, using the same techniques indicated for the analyses of the primary endpoints.

Results of compliance (proportion of presumably consumed medication vs. expected during the considered interval time) will be expressed as a percent and considered as a continuous variable, and as a proportion of subjects with compliance ≥ 75%. The Villalta score [14], which will be analyzed as a continuous variable and as a nominal variable, using the cut-off > 5 to identify the post-thrombotic syndrome and the cut-off > 14 to classify the syndrome as severe.

### Ethics and dissemination

The Jason study is an investigator-initiated trial which is promoted by the “Arianna Anticoagulazione” Foundation (Bologna, Italy). The study is supported by Alfasigma (Bologna, Italy), which also provides all medications. The study is registered at https://www.clinicaltrials.gov (NCT 04257487, registration date February 5th, 2020) and at EudraCT (2019-000570-33). The study protocol [GIASONE (FAAI2.10.2018) V1 date 23th October 2018], was designed in compliance with the current version of the Declaration of Helsinki and the GCP, and was authorized by AIFA (“Agenzia Italiana del Farmaco” ref number AIFA/SC/P95802, date 27th August 2019); it was approved by the Independent Ethics Committee of the Humanitas Research Hospital, Rozzano (Milano), Italy (reference number: 665/19, dated 26th September 2019) as lead ethics committee and will be approved by ethics committees at all participating sites before starting the enrolment. The study will be conducted in accordance with the Declaration of Helsinki. Written informed consent will be collected before starting. Protocol amendments will be communicated to the study sites by the sponsor.

The steering committee (appendix C) has responsibility for the design, conduction and analysis of the study as well as decisions on publication of results and has full access to the data set. All safety and efficacy endpoints will be evaluated by an independent Adjudication Committee (appendix C), blinded to the treatment group, eCRF, e-trial system, data capture and monitoring. A Data Monitoring and Safety Board (DMSB, appendix C), blind to treatment groups, will supervise the study. The trial supporter (Alfasigma, Bologna, Italy) has no role in collection, analysis and interpretation of data, or decision to submit results. Study findings will be disseminated to Alfasigma, to the participating centers, at research conferences, and in peer-reviewed journals.

## Discussion

How to prevent VTE recurrences after a first event in very elderly patients is still an open issue. First of all, the risk of recurrent events in this population is not well defined yet; the risk of the first event is certainly higher in elderly patients; however, the risk of recurrent events does not seem to be higher than that in the general population [20]. Secondarily, while the need of anticoagulation therapy for 3 to 6 months after an acute VTE in elderly patients is unanimously accepted by guidelines and professionals, the indication for an extended therapy is not generally recommended in this population setting [4], mainly for the fear of bleeding complications during anticoagulation beyond the necessary initial and maintainance treatment.

Only a few observational studies and randomized-controlled trials have reported data on bleeding events in elderly patients anticoagulated for VTE. In the observational Worcester Venous Thromboembolism study [21] the incidence rates of major bleeding episodes during VKA treatment in patients aged ≥ 65 years versus those < 65 years were 13.2% and 6.6% respectively after 1 year (*p* < 0.001). In a subsequent analysis of the same cohort, 77% of the bleeding events occurred during anticoagulation with the risk of major bleeding reaching 10.6% after 3 years [22]. In a cohort of 610 very old patients aged ≥ 90 years from the RIETE registry major bleeding complications occurred in 4.9% of them, more than half fatal [23]. In the observational prospective EPICA study the rate of major bleeding events among the 1078 patients aged 80 years or more who started VKA for VTE was 2.4% per year during a median time of 1.8 years [24]. Among the patients aged ≥ 65 years with acute VTE included in the Swiss cohort study SWITCO65 bleeding was the cause of death in 6%, a proportion that remained fairly constant over time (5–8%) [25]. In the management DULCIS study, that included patients after a first unprovoked VTE episode [26], 162 patients aged 75 years or more resumed anticoagulation on the basis of positive D-dimer assays. During the subsequent VKA treatment, the rate of major bleeds was 3.1% per year, whereas it was 1.7% per year in patients aged < 75 years. These data seem to discourage extended anticoagulation with VKA in elderly patients after the first VTE event.

Randomized controlled trials have been conducted to evaluate the efficacy and safety of DOACs versus warfarin in patients with VTE. None of them, however, specifically examined elderly patients, a population that—with some exception—was under-represented in these trials, being around 12–15% of the entire study population. A meta-analysis of these trials reported a risk of bleeding in patients aged ≥ 75 years of 2.1% and of 3.8% in patients treated with a DOAC or warfarin, respectively, [OR 0.56 (*p* = 0.003)], indicating a superior safety profile of DOACs [27]. The reported risk of major bleeding among elderly patients was as low as 1.0% in one trial [28] and ranged from 12.5% to 16.7% when the bleeding risk was reported as the composite of major bleeding and clinically relevant non-major bleeding in other trials [29, 30].

Two trials assessed the efficacy and safety of two doses of a DOAC for extended anticoagulation therapy after a period of standard treatment. In the first (Amplify Extension trial [9]), 5 mg or 2.5 mg apixaban BID were used versus placebo. The rate of recurrent VTE in the whole study population with the 2.5 mg dose was 3.8% versus that of 11.6% in the placebo group, and that of major bleeding events was 0.2% and 0.5%, respectively. In the Einstein Choice trial [10], where 20 mg or 10 mg rivaroxaban OID were compared with aspirin 100 mg OID, the rates of recurrences were 1.2% with 10 mg rivaroxaban treatment and 4.4% in aspirin-treated patients; major bleeding occurred in 0.4% and 0.3%, respectively. Both trials concluded that the lower doses of the tested drugs were suitable for extended treatment in the general population. However, the portion of elderly patients (aged ≥ 75 years) included in the two trials was very small (13.2% and 11.7%, respectively). Furthermore, the results obtained in that segment of the population, both in terms of efficacy and safety, were certainly less good than those recorded in younger patients, and substantially less satisfactory. As for the Amplify Extension trial, the rate of recurrences in elderly patients receiving 2.5 mg dose apixaban was 5.4% versus 12.8% in placebo, and that of major + non-major but clinically relevant bleeding was 6.3% versus 0.9% in placebo (supplemental material). In the Einstein Choice trial, the rate of recurrences in elderly patients receiving 10 mg rivaroxaban was 3.0% versus 4.1% in aspirin-treated patients, and those of major + non-major but clinically relevant bleeding were 1.5% and 5.5%, respectively (supplemental material). The Einstein-Choice results also show that treatment with aspirin 100 mg/day is associated with a non-negligible risk of recurrent events and of bleeding complications and, therefore, is not a good choice for extended treatment in elderly subjects.

In the randomized, placebo-controlled SURVET study [11], the oral administration of sulodexide (500 LSU, BID) for 2 years in patients who had suffered from a first idiopathic VTE and had already undergone an adequate period of anticoagulant therapy, has allowed to reduce the incidence of VTE recurrence by 50% compared to patients treated with placebo, without involving any case of major bleeding. Furthermore, evidence on the absence of the haemorrhagic effect of oral sulodexide administration is already available in the literature [31].

On the basis of the above data, the Jason study is designed to assess whether sulodexide, an antithrombotic agent that does not increase the risk of bleeding when administered orally, can be a useful option in the elderly population to reduce VTE recurrences without giving more bleeding complications. The study will compare the effects of two different doses of sulodexide given for 12 months versus placebo. The choice to test also the dose of 250 LSU BID (one active capsule twice daily), that is half of that used in the SURVET study (500 LSU, BID, two active capsule twice daily), aims at assessing whether an advantage in term of efficacy might be obtained also using less capsules per day in patients often under multiple treatments. Furthermore, that dose is the one the dose that is recommended in the Summary of Product Characteristics of Sulodexide for other clinical indications (e.g. post-thrombotic syndrome). and finally, the daily treatment cost could be reduced.

## Limitations

This is an investigator-initiated trial, and therefore financial resources are spare and should be used thoroughly. This is why periodic on-site monitoring will not be possible. However, a dedicate remote monitor will assure adequate training for the study procedures of all participant investigators before enrolling the first patient. Moreover, the dedicate monitor will check on a regular basis the compliance of sites to protocol, and the complete and correct entry into the eCRF.

## Conclusion

How to treat elderly patients (≥ 75 years) after a first VTE episode, especially if it was unprovoked, is still a controversial issue. Uncertain is the indication for an extended anticoagulant treatment after the first 3 to 6 months. Not only the real VTE recurrence risk is still not clearly defined in that population, but also—and primarily—the risk of bleeding during anticoagulation therapy is higher in elderly than in young patients, so to discourage an extended anticoagulation. Sufficient evidence for that is available for therapy with VKAs. The rather common practice in real-life based on treatment with a low daily dose of acetylsalicylic acid after having discontinued the anticoagulant therapy proved to imply a lower efficacy against relapses than the use of anticoagulants, and—what is of greater concern—to be associated with considerable haemorrhagic risk, especially in the elderly (supplementary data of the Einstein Choice trial [10]). The use of DOACs, especially at doses lower for extended than for initial/long term therapy, is an important option for the general patient population; however, their results are less satisfactory, especially for safety, in elderly patients. The oral administration of the antithrombotic agent Sulodexide proved to significantly reduce the risk of recurrence versus placebo in a general population of VTE patients without increasing the risk of bleeding [11].

The multicenter, randomized, double-blind, placebo-controllod Jason study, will assess the efficacy on the risk of VTE recurrence and safety of oral administration of two doses of Sulodexide Vessel^®^, in a population of elderly patients, with also other risk factors for bleeding, who have accomplished at least three months of anticoagulation therapy after their first VTE event.

## Electronic supplementary material

Below is the link to the electronic supplementary material.

Supplementary file1 (DOCX 29 kb)
