# Supplementary material for: Design and rationale of a randomized, placebo-controlled trial on the efficacy and safety of sulodexide for extended treatment in elderly patients after a first venous thromboembolism
Source: Intern Emerg Med. 2020 May 25;16(2):359–68. doi: 10.1007/s11739-020-02381-5 (PMC7952285; doi:10.1007/s11739-020-02381-5)
Supplement: Supplementary file 1 — Supplementary file1 (DOCX 29 kb) [file 11739_2020_2381_MOESM1_ESM.docx]

Appendix A

List of participating centers

**Coordinating center:** **Dr. Lodigiani Corrado; Centro Trombosi e Malattie Emorragiche - Istituto Clinico Humanitas - IRCCS Humanitas Research Hospital - Via Manzoni, 56 - 20089 Rozzano (MI) Tel 02-82244656/4602 E-mail corrado.lodigiani@humanitas.it**

1. Prof. Ageno Walter DB Internistica e Centro Trombosi ed Emostasi - AO di Circolo - ASST dei Sette Laghi, Viale Borri, 57 - 21100 Varese Tel 0332 278831 Fax 0332 393640 E-mail walter.ageno@uninsubria.it
2. Dott.ssa Angeloni Lucia Dipartimento di Cure Primarie Ospedale "Dossetti" di Bazzano Distretto di committenza e garanzia Reno, Lavino e Samoggia AUSL Bologna Tel 051-6543715 giov. 051622411 mart 051838801 lu-me-ve E-mail l.angeloni@ausl.bologna.it
3. Dott.ssa Barcellona Doris Struttura Semplice Dipartimentale di Emostasi e Trombosi, Policlinico “Duilio Casula” Azienda Ospedaliero-Universitaria di Cagliari Tel 07051096083

E-mail doris.barcellona@unica.it

1. Dott. Barillari Giovanni SOS di Malattie Emorragiche e Trombotiche, Dipartimento di Area Vasta di Medicina Trasfusionale, Presidio Ospedaliero "S. Maria della Misericordia", Azienda Sanitaria Universitaria Integrata di Udine, Via Pozzuolo, 330 – 33100 Udine Tel 0432554488 Fax 0432552342 E-mail barillari.giovanni@aoud.sanita.fvg.it
2. Dott.ssa Borchiellini Alessandra Centro Di Riferimento Regionale Malattie Trombotiche Ed Emorragiche Del Paziente Adulto- S.C. EMATOLOGIA A.O.U. Citta della Salute e della Scienza di Torino Ospedale Molinette: C.so Bramante, 88/90 - 10126 Torino Tel 011 633 5329 Fax 011 6963737 E-mail aborchiellini@cittadellasalute.to.it
3. Dott. Bottino Fabrizio U.O.C. Medicina e Chirurgia d'accettazione e d'urgenza Presidio Ospedaliero Valdarno "La Gruccia" di Arezzo Piazza Del Volontariato, 2, 52025 Montevarchi AR Tel 055/9106526 E-mail fabrizio.bottino@uslsudest.toscana.it
4. Dott. Bucherini Eugenio S.S. Medicina vascolare - AUSL Romagna Ospedale Civile di Faenza, Viale Stradone 7 - 48018 Faenza (RA) Tel 0546/601495 Fax 0546/601169 E-mail eugenio.bucherini@auslromagna.it
5. Prof.ssa Cacciola Rossella U.O.C. Emostasi "G. Rodolico" A.O.U. "Policlinico-Vittorio Emanuele" via Plebiscito 628 Catania Tel 095-3782265 Fax 095-3781956 E-mail rcacciol@unict.it
6. Dott. Carli Giuseppe UOC Ematologia, Centro Malattie Emorragiche e Trombotiche (CMET) - AULSS 8 Berica Ospedale S. Bortolo - Viale Rodolfi, 37 - 36100 Vicenza Tel 0444 75-3518 Fax 0444 75-3365 E-mail g.carli@aulss8.veneto.it
7. Prof. Cattaneo Marco Medicina 3, ASST Santi Paolo e Carlo, San Paolo - Via Di Rudinì, 8 - 20142 Milano Tel 02 81844638 Fax 2509323089 E-mail marco.cattaneo@unimi.it
8. Dott. Bertini Michele UOC Patologia Clinica Presidio Ospedaliero San Filippo Neri - Asl Roma 1 Via Giovanni Martinotti 20 00135 ROMA Tel 0633062629 – 0633062977 Fax 0633063098 email michele.bertini@aslroma1.it
9. Dott. Chistolini Antonio Sezione Ematologia, Dipartimento di Biotecnologie Cellulari ed Ematologia, Azienda Ospedaliero Universitaria Policlinico Umberto I, Roma Tel 06 49974413 Fax 06 44241984 E-mail antonio.chistolini@uniroma1.it
10. Dott.ssa Colucci Antonietta Ambulatorio Emostasi e Trombosi, Servizio di Immunoematologia e Medicina Trasfusionale Ospedale "Di Venere" Bari, ASL Bari Tel 080/5015999 Fax 080/501522 E-mail antinietta.colucci@asl.bari.it
11. Dott.ssa Cosmi Benilde U.O. di Angiologia e Malattie della Coagulazione - Azienda Ospedaliero Universitaria di Bologna - Policlinico S. Orsola - Malpighi, Via Albertoni, 15 - 40138 Bologna Tel 051 2142483/2301 Fax 051 6362517 E-mail benilde.cosmi@unibo.it
12. Prof. De Cristofaro Raimondo Dipartimento di Oncologia ed Ematologia, Istituto di Medicina Interna e Geriatria, Centro per emorragie e malattie trombotiche ed emofilia, Ospedale "A. Gemelli", Università Cattolica, Roma. Tel 06-30156329 E-mail [raimondo.decristofaro@unicatt.it](mailto:raimondo.decristofaro@unicatt.it)
13. Dott.ssa De Micheli Valeria, U.O.S.D. Ematologia: emostasi e trombosi Dipartimento di Medicina ASST-Lecco, Ospedale “Alessandro Manzoni” Lecco
14. Dott.ssa Duce Rita S.C. Laboratorio di analisi, E.O. Ospedali Galliera, Genova Tel 010-5634106 Fax 010-5634125 E-mail rita.duce@galliera.it
15. Dott.ssa Elmi Giovanna Programma Ecografia - Ospedale Maggiore - Bologna Tel 051 6478663 Fax E-mail giovanna.elmi@ausl.bologna.it
16. Dott.ssa Falanga Anna Divisione di Immunoematologia e Medicina Trasfusionale & Centro Emostasi e Trombosi - ASST Papa Giovanni XXIII - Piazza O.M.S., 1 - 24127 Bergamo Tel 0352675057 Fax 0352674832 E-mail afalanga@asst-pg23.it
17. Dott. Fregoni Vittorio U.O.C. Medicina Generale, Ospedale di Sondalo, ASST della Valtellina e dell'Alto Lario, via Zubiani 33, Sondalo Tel 0342 808312 Fax 0342 808239 E-mail vittorio.fregoni@asst-val.it
18. Dott. Galgano Giuseppe "UOC Cardiologia e UTIC - UOC Oncologia ed Ematologia . Centro Emostasi e Trombosi - Ospedale Generale Regionale “ F. Miulli” - Via Provinciale per Santeramo Km 1 - 70021 Acquaviva delle Fonti ( BA) Tel 080 3054357 Fax 0807580809 E-mail giuseppegalgano@hotmail.com
19. Dott.ssa Grandone Elvira UO Emostasi e Trombosi - Centro Ricerca Poliambulatorio Giovanni Paolo II - IRCCS Ospedale Casa Sollievo della Sofferenza - V.le Cappuccini, 1 - 71013 San Giovanni Rotondo (FG) Tel 0882/416286 Fax 0882/416273 E-mail e.grandone@operapadrepio.it
20. Prof. Gresele Paolo Sezione di Medicina Vascolare e d’Urgenza-Stroke Unit, Dipartimento di Medicina, Azienda Ospedaliera di Perugia Tel 075-5783989 Fax 075-5858439 E-mail paolo.gresele@unipg.it
21. Dott.ssa Grifoni Elisa UOC Medicina Interna II - Ospedale S. Giuseppe, Empoli - Azienda USL Toscana Centro Tel 0571/706245 Fax 0571/706247 E-mail elisa.grifoni@uslcentro.toscana.it
22. Dott. Iotti Matteo "S.C. Medicina Cardiovascolare - Dip. Internistico - AUSL di Reggio Emilia - Arcispedale S. Maria Nuova - V.le Risorgimento, 80 - 42123 Reggio Emilia Tel 0522.296.689 - 296.622- 296.533 E-mail matteo.iotti@ausl.re.it
23. Dott.ssa Lami Francesca "Centro trombosi Estense - Medicina interna d'urgenza - Azienda Ospedaliero Universitaria Policlinico di Modena - Ospedale Civile S. Agostino Estense - Via P. Giardini, 1355 - 41126 Modena Tel 059 3961100 Fax 059 3961419 E-mail lami.francesca2@aou.mo.it
24. Dott. Lessiani Gianfranco Ambulatorio Medicina Vascolare/Angiologia, UO Medicina 2 - casa di Cura Conv. Villa Serena - V.le Petruzzi, 42 - 65013 Città Sant'Angelo (PE) Tel 085.9590414 0859590222 E-mail gf.lessiani@gmail.com
25. Dott.ssa Martinelli Ida Centro Emofilia e Trombosi Angelo Bianchi Bonomi Unità Operativa Complessa di Medicina Generale – Emostasi e Trombosi Fondazione IRCCS Ca’ Granda – Ospedale Maggiore Policlinico di Milano Tel 02 5503 5468 E-mail ida.martinelli@policlinico.mi.it
26. Dott.ssa Mastroiacovo Daniela UOSD Angiologia e Diagnostica Vascolare - Ospedale Civile SS Filippo e Nicola - Via G. di Vittorio, snc, 67051 Avezzano (AQ) Tel 086349932

E-mail daniela.mastroiacovo@gmail.com

1. Dott. Mumoli Nicola UOC Medicina Interna- Ospedale Fornaroli, Via Donatore di sangue, 50 - 20013 Magenta (MI) Tel 0297963365/369 E-mail nicola.mumoli@asst-ovestmi.it
2. Dott. Parisi Roberto UOSD Ipertensione e Patologie Endocrine Metaboliche Angiologiche - Ospedale SS. Giovanni e Paolo, AULSS3 Serenissima - Via Castello, 6777 - 30122 Venezia Tel 0415294209/5544 E-mail roberto.parisi@aulss3.veneto.it
3. Dott. Pesavento Raffaele U.O.C. Clinica Medica 3, Azienda Ospedaliera di Padova

Tel 0498218732 E-mail raffaele@weightwind.org

1. Prof. Pignatelli Pasquale "UOC Di Medicina Interna e Prevenzione Dell’Aterosclerosi “Policlinico Umberto I - Viale del Policlinico 155 - 00163 Roma Tel 0649977777 E-mail pasquale.pignatelli@uniroma1.it
2. Dott.ssa Poli Daniela SOD Malattie Aterotrombotiche, AOU-Careggi, Largo G. Alessandro Brambilla, 3, 50134 Firenze Tel 0557945509 E-mail daniela.poli.mail@gmail.com polida@aou-careggi.toscana.it
3. Dott. Porfidia Angelo "UOC Medicina Generale Fondazione Policlinico Universitario Agostino Gemelli IRCCS" Tel 06.3015.7597 Fax 06.35502775 E-mail angelo.porfidia@policlinicogemelli.it
4. Dott. Prior Manlio UOC Angiologia - Azienda Ospedaliera Universitaria Integrata di Verona Borgo Roma, Piazzale L. A. Scuro, 10 Tel 045 8126808 Fax 045 8126806 E-mail manlio.prior@univr.it
5. Dott. Rescigno Giuseppe SC Laboratorio patologia clinica Centro sorveglianza TAO Presidio Ospedaliero Umberto I Nocera inferiore ASL Salerno Ospedale Tortora Di Pagani (SA) Tel 0819213558 – 3545 Fax 0819213558 E-mail g.rescigno1@gmail.com
6. Dott. Ria Luigi "U.O. di Medicina Interna, Ospedale ""S. Cuore di Gesù"" Gallipoli (Lecce) via Alezio Gallipoli 73014 Lecce" Tel 0833-270717 Fax 0833-270706 E-mail luigiria@tiscali.it
7. Dott. Paolo Chiarugi "Ambulatorio Antitrombosi - U.O Laboratorio Analisi Chimico Cliniche Azienda Ospedaliero-Universitaria Pisana - Nuovo S. Chiara - Via Paradisa, 2 - 56124 Pisa " Tel 050-995592 E-mail p.chiarugi@ao-pisa.toscana.it
8. Dott.ssa Rupoli Serena SOD Clinica Ematologica Azienda Ospedaliero Universitaria Ospedali Riuniti Umberto I di Ancona Via Conca, 71 - 60126 Ancona Tel 071-5964771 Fax 071-5964748 E-mail serena.rupoli@ospedaliriuniti.marche.it
9. Prof. Santoliquido Angelo UOS Angiologia Columbus Policlinico Gemelli Tel 06-3015.9854

E-mail [angelo.santoliquido@policlinicogemelli.it](mailto:angelo.santoliquido@policlinicogemelli.it)

1. Prof. Sartori Maria Teresa Clinica Medica 1 Dipartimento Di Medicina Azienda Ospedaliera Universitaria Padova Via N. Giustiniani 2 35100 Padova Tel. 049 8212165 - 049 8212653

Fax 049 8212159 email [mtsart@unipd.it](mailto:mtsart@unipd.it)

1. Dott.ssa Testa Sophie UUOO Laboratorio Analisi chimico-cliniche e microbiologiche - ASST Cremona - V.le Concordia, 1, 26100 Cremona Tel 0372 405452 E-mail s.testa@asst-cremona.it
2. Dott.ssa Villalta Sabina Medicina Interna 1 - Ospedale Cà Foncello - AULSS2 Marca Trevigiana - Piazza Ospedale, 1 - 31100 Treviso Tel 0422 322230 Fax 0422 322314 E-mail sabina.villalta@aulss2.veneto.it
3. Dott.ssa Visonà Adriana "UOC Angiologia - Dipartimento di Medicina Clinica - Azienda ULSS 2 Marca Trevigiana - Ospedale San Giacomo Apostolo - Via dei Carpani 16/Z - 31033 Castelfranco Veneto (TV) Tel 0423 732912 E-mail [adrianavisona@gmail.com](mailto:adrianavisona@gmail.com)
4. Dott. Zanatta Nello UOSD di Angiologia - Ospedale di Conegliano - Via Brigata Bisagno, 4 - 31015 Conegliano (TV) Tel 0438663356 E-mail nello.zanatta@aulss2.veneto.it
5. Dott. Ziliotti Maurizio U.O. Medicina Interna P.O. Vaio, AUSL Parma - Via Don Tincati, 5 - 43036 Fidenza (PR) Tel 0524 515855 E-mail mziliotti@ausl.pr.it
6. Dott.ssa Zingaretti Oriana SOSD Medicina Vascolare - Ospedali riuniti - Torrette (AN) Tel 071 5965732 Fax 071 596 4670 E-mail [oriana.zingaretti@ospedaliriuniti.marche.it](mailto:oriana.zingaretti@ospedaliriuniti.marche.it)
7. Dott.ssa Pancani Roberta U.O. Pneumologia Universitaria Azienda Ospedaliero-Universitaria Pisana - Cisanello - Pisa Tel. 050.995360/05099539 E-mail [frapanca@tin.it](mailto:frapanca@tin.it)
8. Dott.ssa Marconi Letizia U.O Medicina Interna della Lunigiana Presidio Ospedaliero di

Appendix B

STUDY COMMITTEES

***1. Steering Committee (SC)***:

a. supervises the course of the study and ensures that the protocol is carefully followed by each participant. To exert this responsibility, the Steering Committee is supported by the Monitor(s); is informed of all the monitoring activities and receives – and should take into appropriate consideration – the reports of the Data Monitoring and Safety Board as detailed below;

b. proposes amendments to the protocol to resolve, clarify and implement potential doubts, questions or practical difficulties that may onset during the study;

c. proposes amendments to the protocol that may become necessary upon the scientific progress in the specific therapeutic segment, to ensure the maintenance of the scientific validity of the study;

d. solves doubts concerning possible protocol violations, deciding whether individual cases have to be classified as minor deviation, major deviation or violation proper and, consequently, whether the specific subject is to be included into the safety population only or also into the mITT population or even into the PP population. To this aim, the Steering Committee will have anonymous and rigorously blinded access to all data relevant to the specific case;

e. participates to the Writing Committee;

f. assists the Adjudication Committee in case of irreconcilable disagreement among the members of such Committee in expressing their judgement.

The Steering Committee is composed of Prof. G. Palareti (president), Prof. Walter Ageno, Dott.ssa Benilde Cosmi, Dott.ssa Cristina Legnani, Dott. Corrado Lodigiani, Dott.ssa Ida Martinelli, Prof. Paolo Prandoni, Prof. Vittorio Pengo, Dott.ssa Daniela Poli, Dott.ssa Sophie Testa

***2. Adjudication Committee (AC):***

The Adjudication Committee is responsible of validating the efficacy and safety outcomes reported by the Investigators, and of classifying the doubtful event of recurrence and/or bleeding as outcome or non-outcome. The evaluation by the Adjudication Committee will be blind, with blinded access to all data available onto the database for the specific case. The data will be completely anonymous.

If considered necessary, the Adjudication Committee has the right to request additional information, not available onto the database, to the Investigator responsible for the case. All data supplied to the Adjudication Committee will anyway be completely anonymous or, where necessary, anonymized before being conferred to the Committee. The Investigators participating to the study, for the simple fact of accepting this protocol also accept the possibility of receiving such requests for additional information and accept to supply what requested, if possible and without prejudice for the respect of confidentiality.

The judgement of the Adjudication Committee is taken by consensus among its member. In case of irreconcilable disagreement, the judgement will be deferred to a joint meeting with the Steering Committee, in which the decision will be taken by the majority.

The Adjudication Committee is composed of the Doctors: Prof. Domenico Prisco (Florence), Dr Daniele Imberti (Piacenza) and Dr Franco Piovella (Pavia).

***3. Data Monitoring and Safety Board***

This study will be supervised by a specifically instituted Data Monitoring and Safety Board (DMSB). The main aim of the DMSB is the patients’ protection, in first instance of those enrolled in the study, but also of other patients with the examined disorder. The second responsibility of the DMSB is to ensure the integrity of the study, in relation to the compliance with procedures indicated in the protocol to guarantee the data credibility.

The DMSB is composed of the Doctors Angelo Bignamini (Milan), Alberto Tosetto (Vicenza), and Lorenza Bertù (Varese).

To exert its function the DMSB performs periodical evaluations on the data, writes a report with its recommendations and submits the recommendations to the Steering Committee. These reports are not binding, however, in case the Steering Committee decides not to implement decisions recommended by the DMSB, this latter is free to communicate its recommendation to the Ethics Committees who approved the protocol.

*3.1. Procedures*

The DMSB periodically receives from the study monitor a detailed evaluation of:

- frequency of primary events

- frequency of serious adverse events

- frequency of treatment interruptions

- frequency of subjects lost to follow-up without having incurred into a primary event

- frequency of potential protocol violations.

The evaluations presented to the DMSB will, in principle, not be stratified by treatment group. Only when the classification by treatment group could imply a decision to interrupt the study or the suggestion of a major protocol modification, the results will be stratified by treatment group, always blinded as to which treatment. Unblinding, with the attending consequences under the statistical and procedural viewpoint, will occur only in the case the DMSB intends to recommend the interruption of one study arm only or to recalculate the sample size (with the need to provide a new randomization for the following cases).

Based on these evaluations, the DMSB decides by the majority which recommendation shall be sent to the Steering Committee: continue the study as planned in the protocol; implement further verifications of adequacy and quality of the applied procedures (to decrease the risks of protocol violations and/or facilitate maintaining in the study the recruited patients); interrupt the study for manifest superiority or manifest inferiority or futility; interrupt one of the study arms because of the of indicated reasons; modify the sample size of the whole study or of one of the arms.

*3.2. Interim analyses*

In this study, the periodic assessments will be performed when reaching the first among the following conditions:

a) 30% and 60% of concluded cases, i.e., when 435 and, respectively, 870 patients will have reached the end of the observation, regardless of the number of events, or

b) 30% and 60% of the primary events (composite of recurrence of proximal DVT, new PE episodes, total mortality attributable to TEEs), i.e., when 42 and, respectively, 84 primary events will have been confirmed by the Adjudication Committee.

*3.3 Impact of interim analyses on the alpha error*

Performing two interim analyses in addition to the final analysis implies an increase of the alpha error that, estimated with the O’Brien-Fleming function, imposes to decrease the nominal alpha value for the final analysis from 0.05 to 0.048 (two-tailed) for the primary efficacy endpoint. This correction was already considered in the sample size calculation.

Similarly, the critical alpha value (one-sided) to be considered for the final analysis of the safety endpoint will change from 0.05 to 0.0465 for the effect of the interim analyses. This correction as well was already considered in the sample size calculation.

*3.4. DMSB decisional criteria*

*3.4.1. Interruption due to manifest superiority*

“Manifest superiority” means that one of the groups (treated regardless of dose, or controls) showed at one of the interim analyses a rate of the composite primary efficacy endpoint so largely superior compared with the other group, that continuing the study as planned will have very small probability to change the result. Furthermore, this result invalidates the principle of equipoise among treatments, making unethical the continuation of the study.

Since the considered hypothesis was two-tailed, the criterion to suggest interrupting the study for manifest superiority is that the analysis yields a Z-test value of 3.929 or more at the first interim analysis, or of 2.670 or more at the second interim analysis.

*3.4.2. Interruption due to manifest inferiority*

“Manifest inferiority” means that one of the groups (treated regardless of dose, or controls) showed at one of the interim analyses a rate of bleedings so largely greater compared with the other group, that continuing the study as planned will have very small probability to change the result. Furthermore, this result exposes to unacceptable risks one of the groups, making unethical the continuation of the study.

Since the considered hypothesis was one-tailed, the criterion to suggest interrupting the study for manifest inferiority is that the analysis yields a Z-test value of 3.393 or more at the first interim analysis, or of 2.281 or more at the second interim analysis.

*3.4.3. Interruption due to futility*

“Futility” means that the data collected at the time of the analysis suggest that the study as planned will have little probability of reach its primary objectives. This condition can occur because the rate of events among controls is less than expected and/or the risk reduction among treated is less than expected. If at the first interim analysis the number of events among controls is 14 or less and, at the same time, the number of events among treated (regardless of dose) is 19 or more, in absence of factor that could justify such deviations from the expected, continuing the study will have very small probability of reaching the planned objectives. The same conclusion will be reached if, at the second interim analysis, the number of events among controls will be 28 or less and, at the same time, the number of events among treated (regardless of dose) will be 37 or more.

*3.4.4. Alternative decisions*

In case one of the interruption criteria will be reached, or even without reaching one of such criteria but in presence of deviations considered relevant by the DMSB in relation to the study objectives, the Committee could require that the interim analysis is repeated, stratifying the results by treatment group. In case one group presents sufficient evidence of deviating from expected, the DMSB could suggest to the Steering Committee:

- to recalculate the sample size based on the effectively observed incidence of events;

- to interrupt one the treatment arms based on the deviation of the incidence of events actually observed vs. that expected;

- a combination of both the above suggestions.

***4. Writing Committee***

The Writing Committee is composed of the Steering Committee, complemented with one Investigator indicated by each center having recruited at least 24 patients.

The Writing Committee:

a. prepares the publication of the main study results;

b. decides additional and subsequent publications of planned and unplanned study results;

c. requests additional statistical analyses of study data in relation to the publications indicated above;

d. defines the list of authors for each publication;

e. manages the contacts with the journals and the responses to possible queries for clarifications and modifications to the publications
